# Sensitization of multidrug-resistant cancer cells to Hsp90 inhibitors by NSAIDs-induced apoptotic and autophagic cell death

**DOI:** 10.18632/oncotarget.24130

**Published:** 2018-01-10

**Authors:** Hyun-Jung Moon, Hak-Bong Kim, Su-Hoon Lee, So-Eun Jeun, Chi-Dug Kang, Sun-Hee Kim

**Affiliations:** ^1^ Department of Biochemistry, Pusan National University School of Medicine, Yangsan 626-870, Korea

**Keywords:** Hsp90 inhibitor, NSAID, MDR, autophagy, apoptosis

## Abstract

NSAIDs (non-steroidal anti-inflammatory drugs) have potential use as anticancer agents, either alone or in combination with other cancer therapies. We found that NSAIDs including celecoxib (CCB) and ibuprofen (IBU) significantly potentiated the cytotoxicity of Hsp90 inhibitors in human multidrug-resistant (MDR) cells expressing high levels of mutant p53 (mutp53) protein and P-glycoprotein (P-gp), and reversed Hsp90 inhibitor resistance caused by activation of heat shock factor 1 (HSF1) and by up-regulation of heat shock proteins (Hsps) and P-gp. Inhibition of Akt/mTOR and STAT3 pathways by CCB induced autophagy, which promoted the degradation of mutp53, one of Hsp90 client proteins, and subsequently down-regulated HSF1/Hsps and P-gp. Inhibition of autophagy prevented mutp53 degradation and CCB-induced apoptosis, and inhibition of caspase-3-mediated apoptotic pathway by Z-DEVD-FMK did not completely block CCB-induced cell death in MDR cells, suggesting that autophagic and apoptotic cell death may contribute to CCB-induced cytotoxicity in MDR cells. Furthermore, CCB and IBU suppressed Hsp90 inhibitor-induced HSF1/Hsp70/P-gp activity and mutp53 expression in MDR cells. Our results suggest that NSAIDs can be used as potential Hsp90 inhibitor chemosensitizers and reverse resistance of MDR cells to Hsp90 inhibitors via induction of apoptosis and autophagy. These results might enable the use of lower, less toxic doses of Hsp90 inhibitors and facilitate the design of practically applicable, novel combination therapy for the treatment of MDR cancer.

## INTRODUCTION

Selective inhibition of target proteins in cancer cells over their normal counterparts is one of the most critical features of a successful protein inhibitor for clinical applications. The molecular chaperone heat-shock protein 90 (Hsp90) is constitutively overexpressed or activated in cancer cells than normal cells and therefore provides an attractive target for the treatment of cancer [1]. Since inhibition of Hsp90 simultaneously interrupts many signal transduction pathways that are pivotal to tumor progression and survival through degradation of large numbers of oncogenic client proteins associated with hallmarks of cancer, drugs aimed at inhibiting Hsp90 have been suggested to be active against many types of cancer [2, 3]. Evidence of the activities of Hsp90 inhibitors has been obtained *in vitro* and in animal models, and numerous clinical trials (phase I-III) have been conducted to develop novel cancer treatments [2–5]. A number of phase II clinical trials have been performed on 17-allylamino-17-demethoxy-geldanamycin (17-AAG; a geldanamycin analog) and NVP-AUY922 (hereafter called AUY922; a purine-scaffold derivative and non-geldanamycin analog of 17-AAG) [6–9]. However, their therapeutic benefits were often limited by toxicity and resistance of cancer cells. It has been reported that resistance to Hsp90 inhibitors is linked to P-glycoprotein (P-gp)-mediated efflux and to the induction of heat shock proteins (Hsps) [10, 11], which is caused by the disruption of Hsp90 with heat shock factor 1 (HSF1) complexes and consequent HSF1-mediated induction of cytoprotective Hsps such as Hsp70 and Hsp27 [12].

Mutant p53 (mutp53) protein is often overexpressed in tumors because it escapes proteolytic degradation and consequently has a longer half-life than wild-type p53 (wtp53) protein, which has an extremely short half-life [13]. A high level of mutp53 is known to be related to greater aggressiveness and resistance to therapy and poorer outcomes in some tumors [14, 15]. Mutp53 is an important determinant of HSF1, a major transcription factor for Hsps. Mutp53 facilitates recruitment of HSF1 to specific DNA sites of heat shock elements in target gene promoters and subsequently augments pro-survival HSF1-induced transcriptional program, including expression of Hsps [10]. Inhibition of Hsp90 has been shown to promote the degradation of mutp53, a client protein of Hsp90 [16]. Therefore, Hsp90 inhibitors may be more effective in cancer cells with mutp53 than those with wtp53. Moreover, mutp53 contributes to the transcriptions of multidrug resistant 1 (*MDR1*) gene through cooperation with ETS-1 [17] as well as activation of HSF1 [18]. We previously reported that human MDR cells are highly resistant to Hsp90 inhibitors as compared with their parental cells, and that knockdown of HSF1 in MDR cells induced the down-regulation of mutp53 and subsequently prevented the P-gp-mediated efflux of Hsp90 inhibitor [19]. Therefore, we hypothesized that combined treatment of Hsp90 inhibitor and novel HSF1/mutp53 modulator might enhance the anticancer activity of Hsp90 inhibitors and reverse resistance to Hsp90 inhibitors in MDR cells.

Non-steroidal anti-inflammatory drugs (NSAIDs) are the most frequently consumed drugs worldwide and they are very effective in the alleviation of pain, fever and inflammation [20]. In addition to these effects, NSAIDs have been shown to reduce cancer cell proliferation, motility, angiogenesis and invasiveness in a wide variety of cancer types [21–23]. The antitumor activity of celecoxib (CCB), one type of NSAIDs, is thought to be associated with its ability to induce apoptosis in a variety of cancer cells [24, 25]. Ibuprofen (IBU), a relatively non-toxic and widely used NSAID, significantly decreased the expression of Hsp70, which mediates the sensitivity to cisplatin by enhancing apoptosis in lung cancer cells [26]. Moreover, several studies have indicated that NSAIDs may sensitize cancer cells to the antiproliferative effects of cytotoxic drugs by down-regulation of ATP-binding cassette transport proteins (ABC transporters) [27–30].

The present study shows that NSAIDs such as CCB and IBU significantly potentiate the sensitivity of MDR cells to Hsp90 inhibitors via the autophagic degradation/down-regulation of mutp53 achieved by inhibiting the Akt/mTOR pathway and STAT3/HSF1/P-gp signaling cascades, suggesting that the clinically approved NSAIDs can be used to increase the overall efficacies and utilities of Hsp90 inhibitors through inhibiting the resistance of cancer cells to Hsp90 inhibitors.

## RESULTS

### Enhanced susceptibility of multidrug-resistant human cancer cells to Hsp90 inhibitors by NSAIDs

**Figure 1:**
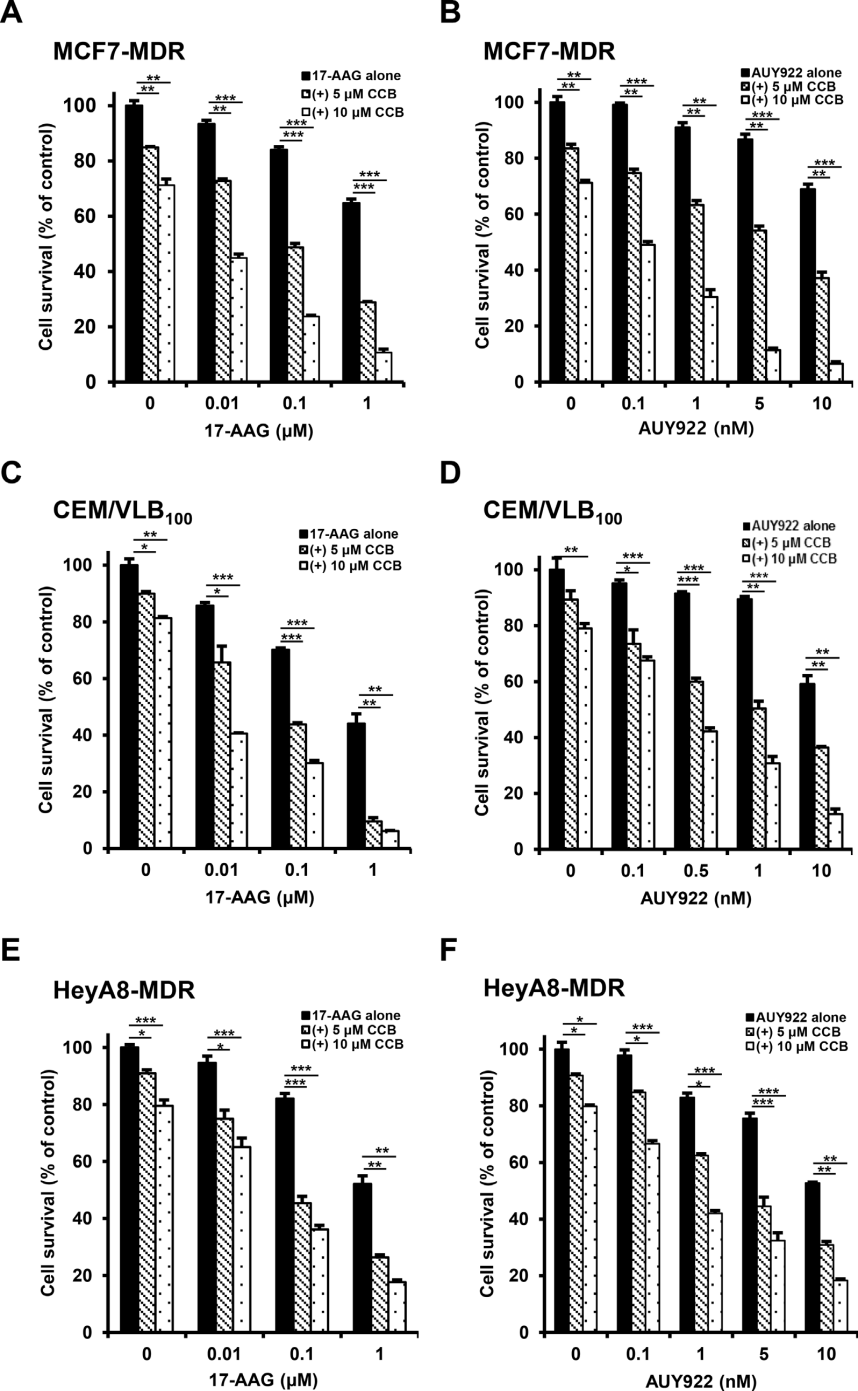
Potentiation of Hsp90 inhibitor-induced cytotoxicity by celecoxib (CCB) in MDR cells MCF7-MDR (**A** and **B**), CEM/VLB_100_ (**C** and **D**) or HeyA8-MDR cells (**E** and **F**) were treated with increasing doses of 17-AAG or AUY922 in the presence or absence of CCB (5 and 10 μM). Percentage of cell survival was determined after 96 h of incubation using the MTT assay. Results are the means ± SEs of three experiments.^*^ *p <* 0.05, ^**^p < 0.01 and ^***^p < 0.001.

**Figure 2:**
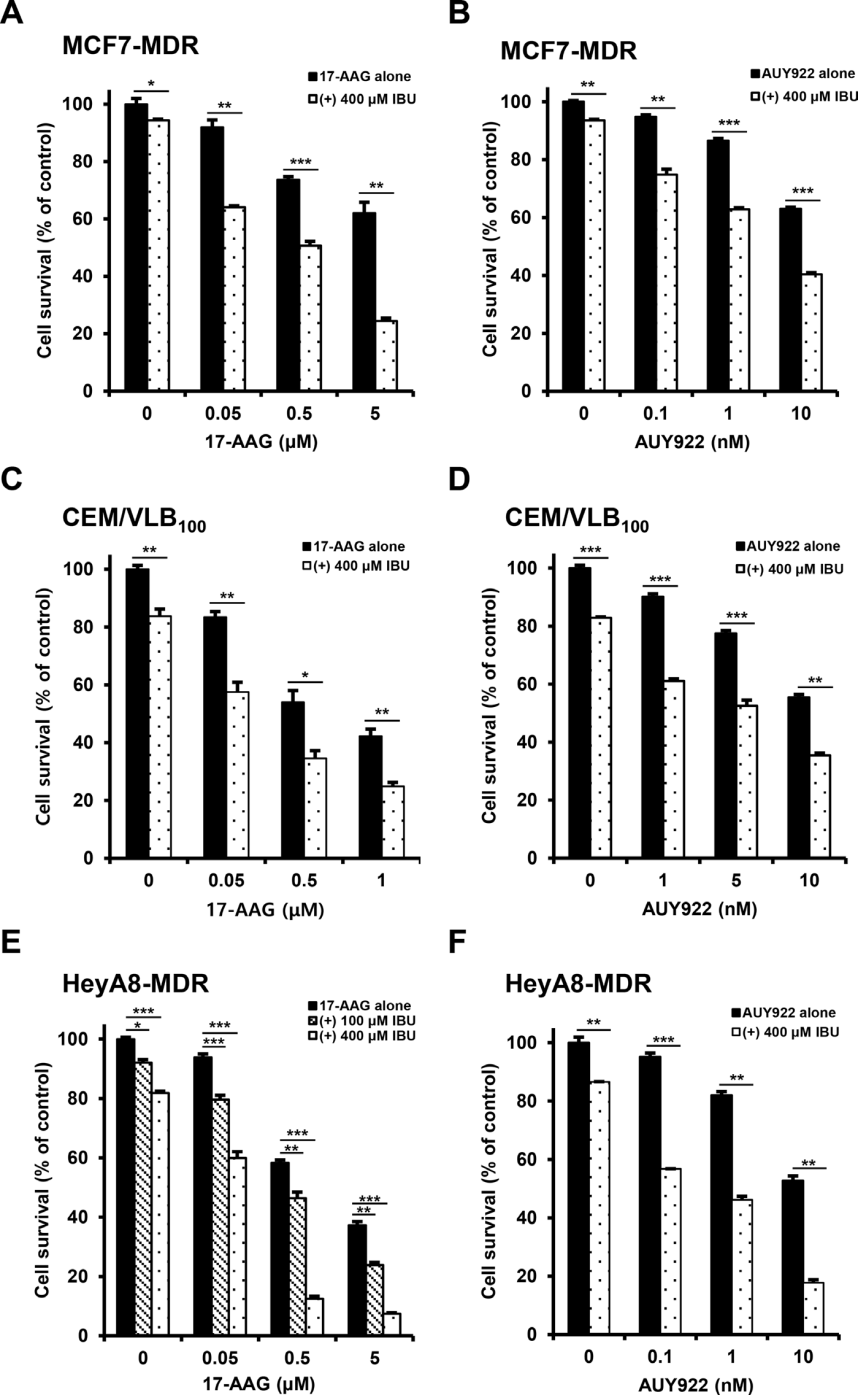
Potentiation of Hsp90 inhibitor-induced cytotoxicity by ibuprofen (IBU) in MDR cells MCF7-MDR (**A** and **B**), CEM/VLB_100_ (**C** and **D**) or HeyA8-MDR cells (**E** and **F**) were treated with increasing doses of 17-AAG or AUY922 in the presence or absence of IBU (100 or 400 μM). Percentage of cell survival was determined after 96 h of incubation using the MTT assay. Results are the means ± SEs of three experiments.^*^ *p* < 0.05, ^**^p < 0.01 and ^***^p < 0.001.

We previously reported that multidrug-resistant (MDR) human cancer cells were significantly more resistant to Hsp90 inhibitors than their parental drug sensitive cells, and resistance to Hsp90 inhibitors in MDR cells could be overcome through down-regulation of Hsps and P-gp [19]. Since some commonly used NSAIDs possessed P-gp modulator activity [28], we investigated whether NSAIDs could act as a new class of chemosensitizers for Hsp90 inhibitor-resistant MDR cells. To evaluate the potential role of NSAIDs in the response of MDR human cancer cells to Hsp90 inhibitors, three different MDR cancer cell lines were treated with gradual doses of 17-AAG, a geldanamycin analog Hsp90 inhibitor or AUY922, non-geldanamycin Hsp90 inhibitor, alone or combined with celecoxib (CCB) and determined whether CCB could enhance the susceptibility of MDR cells to Hsp90 inhibitors using MTT assay. When we tested the combination effect of Hsp90 inhibitors with CCB in MCF7-MDR cells, MDR variant of human breast cancer MCF-7 cells, CCB significantly enhanced the susceptibility of MCF7-MDR cells to both 17-AAG and AUY922 (Figure 1A and 1B). Average combination index (CI) values for combination treatments with CCB and 17-AAG (or AUY922) were lower than 0.5 at all concentrations, indicating a synergistic effect in MCF7-MDR cells. Similar result was observed in other MDR cell lines, namely, CEM/VLB_100_ cells, MDR variant of CEM human T-lymphoblastic leukemia cells (Figure 1C and 1D), and HeyA8-MDR cells, MDR variant of HeyA8 human ovarian cancer cells (Figure 1E and 1F). When CEM/VLB_100_ and HeyA8-MDR cells treated with 17-AAG or AUY922 alone or combined with CCB, the growth-inhibitory effect of 17-AAG or AUY922 combined with CCB was greater than that of 17-AAG (or AUY922) alone, and average CI values for combination treatment with Hsp90 inhibitor and CCB were lower than 0.5 at all concentrations, indicating a synergistic effect of Hsp90 inhibitor and CCB in two other MDR cell lines. These observations suggest that CCB could potentiate the sensitivity of MDR cells to Hsp90 inhibitors. To more confirm the combination effect of another NSAID and Hsp90 inhibitor against MDR cells, we examined whether ibuprofen (IBU) could enhance the cytotoxic effect of Hsp90 inhibitor in MDR cells. Similarly, enhancement of 17-AAG (or AUY922)-induced cytotoxicity by IBU treatment was observed MCF7-MDR (Figure 2A and 2B), CEM/VLB_100_ (Figure 2C and 2D) and HeyA8-MDR cells (Figure 2E and 2F). In these three MDR lines, combination treatment of IBU and 17-AAG (or AUY922) induced synergistic growth inhibition, with CI value as low as 0.9 at any concentration tested. These results suggest that susceptibility of MDR cells to Hsp90 inhibitors might be enhanced by NSAIDs, and therefore NSAIDs could act as chemosensitizing agents for Hsp90 inhibitor-resistant MDR cells.

### Down-regulation of mutp53 protein in MDR cells by NASIDs

**Figure 3:**
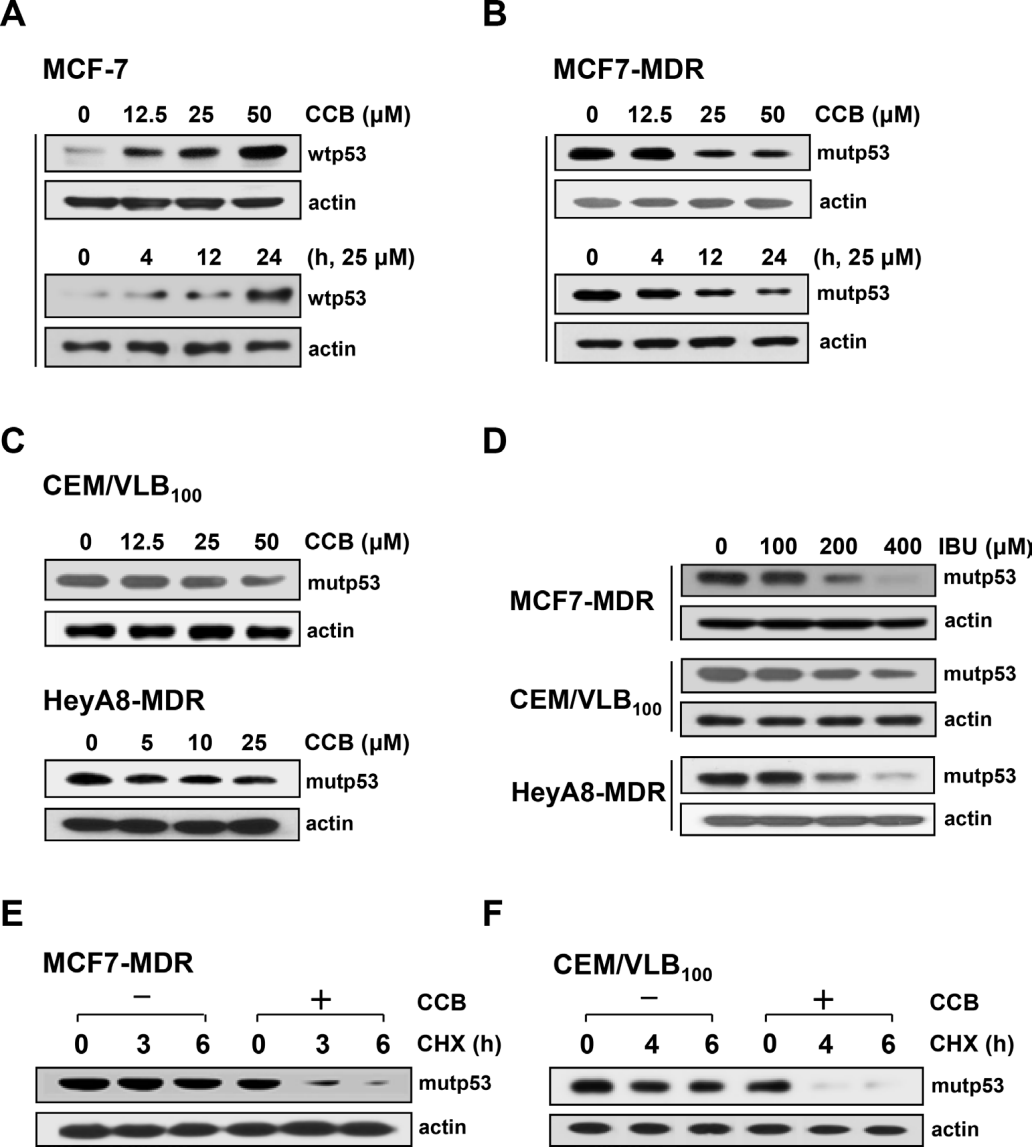
Reduction of mutp53 protein levels in MDR cells by NSAIDs MCF-7 (**A**) and MCF7-MDR cells (**B**) were treated with increasing doses of CCB for 24 h or with 25 μM CCB for the indicated times, and the levels of wild-type (wtp53) and mutant p53 (mutp53) proteins were determined by western blot analysis and. Changes in mutp53 level in CEM/VLB_100_ or HeyA8-MDR cells treated with increasing doses of CCB for 24 h (**C**) and in three MDR cell lines treated with increasing doses of IBU for 24 h (**D**) were determined by western blot analysis. MCF7-MDR (**E**) or CEM/VLB_100_ cells (**F**) were treated with cycloheximide (CHX, 20 μg/ml for 3∼6 h) in the absence or presence of 25μM CCB. Changes in mutp53 levels were determined by western blot analysis. β-Actin (actin) was used as a loading control.

P-glycoprotein (P-gp), *MDR1* gene product, confers multidrug resistance against antineoplastic agents but also contributes in part to acquired resistance to some Hsp90 inhibitors [12]. It has been reported that mutp53 protein, one of important client proteins of Hsp90, up-regulated the *MDR1* promoter and thus positively regulated P-gp [17]. To address whether treatment of MDR cells with CCB specifically targets down-regulation of mutp53, we investigated the differential effect of CCB on MCF-7 cells carrying wild-type p53 (wtp53) protein and MCF7-MDR cells carrying mutp53. Treatment of MCF-7 cells with CCB resulted in a dose- and time-dependent up-regulation of wtp53 (Figure 3A), whereas MCF7-MDR cells treated with CCB showed a dose- and time-dependent down-regulation of endogenous mutp53 protein levels under the same treatment conditions (Figure 3B), indicating selective down-regulation of mutp53 but not of wtp53 by CCB. Similarly, the expression of mutp53 was significantly reduced by CCB treatment in CEM/VLB_100_ and HeyA8-MDR cells (Figure 3C). Moreover, in the three MDR cell lines, the level of mutp53 was significantly reduced by IBU treatment (Figure 3D), indicating the possible involvements of mutp53 down-regulation in MDR cells by NSAIDs. Next, to examine whether CCB down-regulated mutp53 through post-translational degradation, changes in levels of mutp53 protein in MCF7-MDR and CEM/VLB_100_ cells were determined in the presence of cycloheximide (CHX), a protein synthesis inhibitor, after treatment with CCB. Mutp53 level in MCF7-MDR cells was slightly reduced after treatment with CHX alone for 3 h but markedly reduced after co-treatment with CHX and CCB for 3 h (Figure 3E). Similarly, mutp53 levels in CEM/VLB_100_ cells were reduced to undetectable levels by co-treatment with CHX and CCB for 4 h, whereas mutp53 level in cells treated with CHX alone was only slightly reduced after treatment for 6 h (Figure 3F). These results suggest that CCB reduces the half-life of mutp53 in MDR cells possibly by degrading mutp53 protein, and thus, down-regulating mutp53.

### Mutp53 degradation by NASIDs-induced autophagy through COX-2 inhibition and COX-2-unrelated pathways

**Figure 4:**
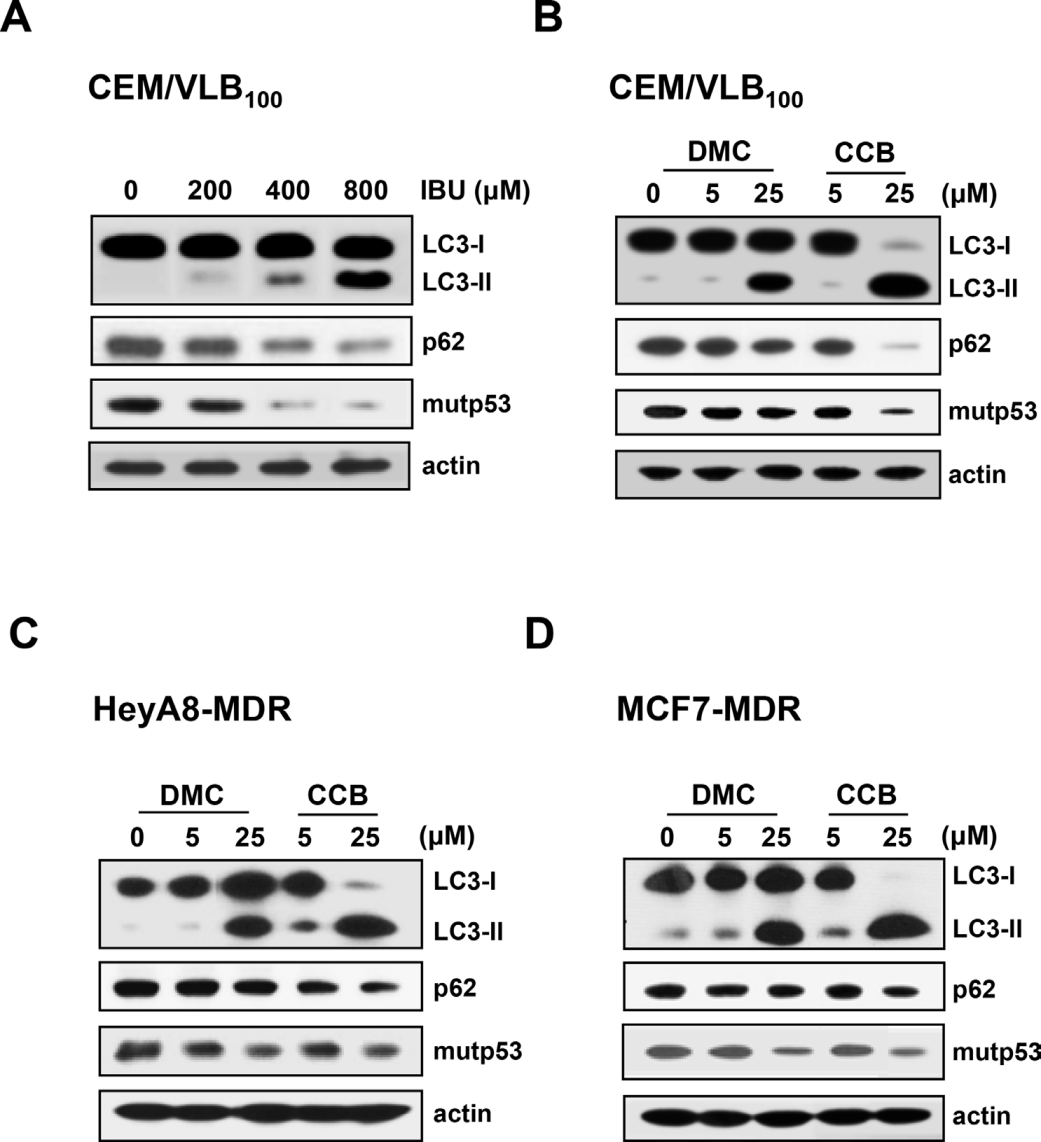
Induction of autophagy and degradation of mutp53 in MDR cells by NSAIDs CEM/VLB_100_ (**A** and **B**), HeyA8-MDR cells (**C**) or MCF7-MDR (**D**) were treated with IBU (200∼800 μM), 2,5-dimethyl-celecoxib (DMC, 5 or 25 μM), or CCB (5 or 25 μM) for 24 h, respectively. The levels of LC3-I/II, p62 and mutp53 were determined by western blot analysis. Actin was used as a loading control.

Since it has been reported autophagy is a key regulator of mutp53 stability [13], we considered that NASID-induced mutp53 degradation might depend on autophagy. To clarify the role of autophagy in the instability of mutp53 induced by NSAIDs, we assessed the effects of NSAIDs on the autophagy-mediated degradation of mutp53. One approach is to detect LC3 conversion (LC3-I to LC3-II) by immunoblot analysis because the amount of LC3-II is clearly correlated with the number of autophagosomes, and an increase in endogenous LC3-II may be regarded as a marker for autophagy [31]. In addition, p62 is known to be incorporated into autophagosomes and efficiently degraded when autophagy is induced, thus p62 is also an important marker for the induction of autophagy [32]. Therefore, we evaluated the autophagic degradation of mutp53 in MDR cells by assessing changes in the levels of LC3-II and p62. When LC3 conversion and p62 level were assessed in IBU-treated CEM/VLB_100_ cells, treatment with IBU dose-dependently enhanced LC3-II levels but reduced p62 levels. In parallel with the autophagy-inducing effect of IBU, the level of mutp53 was reduced in MDR cells (Figure 4A), suggesting IBU-mediated autophagic degradation of mutp53. It is well known that CCB, a cyclooxygenase-2 (COX-2) specific inhibitor, has several COX-2 independent activities [33], and 2, 5-dimethyl-celecoxib (DMC), a close structural analog of the CCB, lacks COX-2 inhibitory function [34] whereas IBU inhibits COX-2 and COX-1 enzymes [35]. Our data showed both DMC and CCB could induce autophagy in various MDR cells. The level of LC3-II was significantly elevated but p62 level was conversely reduced in DMC-treated CEM/VLB_100_ cells, and DMC also reduced mutp53 levels, indicating DMC can induce autophagy despite an inability to block COX-2 (Figure 4B). Autophagy induction and degradation of mutp53 by DMC, nonetheless, was lower compared with those of CCB-treated MDR cells including HeyA8-MDR cells (Figure 4C) and MCF7-MDR cells (Figure 4D). These results suggest that NSAIDs-induced autophagy and degradation of mutp53 in MDR cells occur through both COX-2 inhibition and COX-2-unrelated pathway.

**Figure 5:**
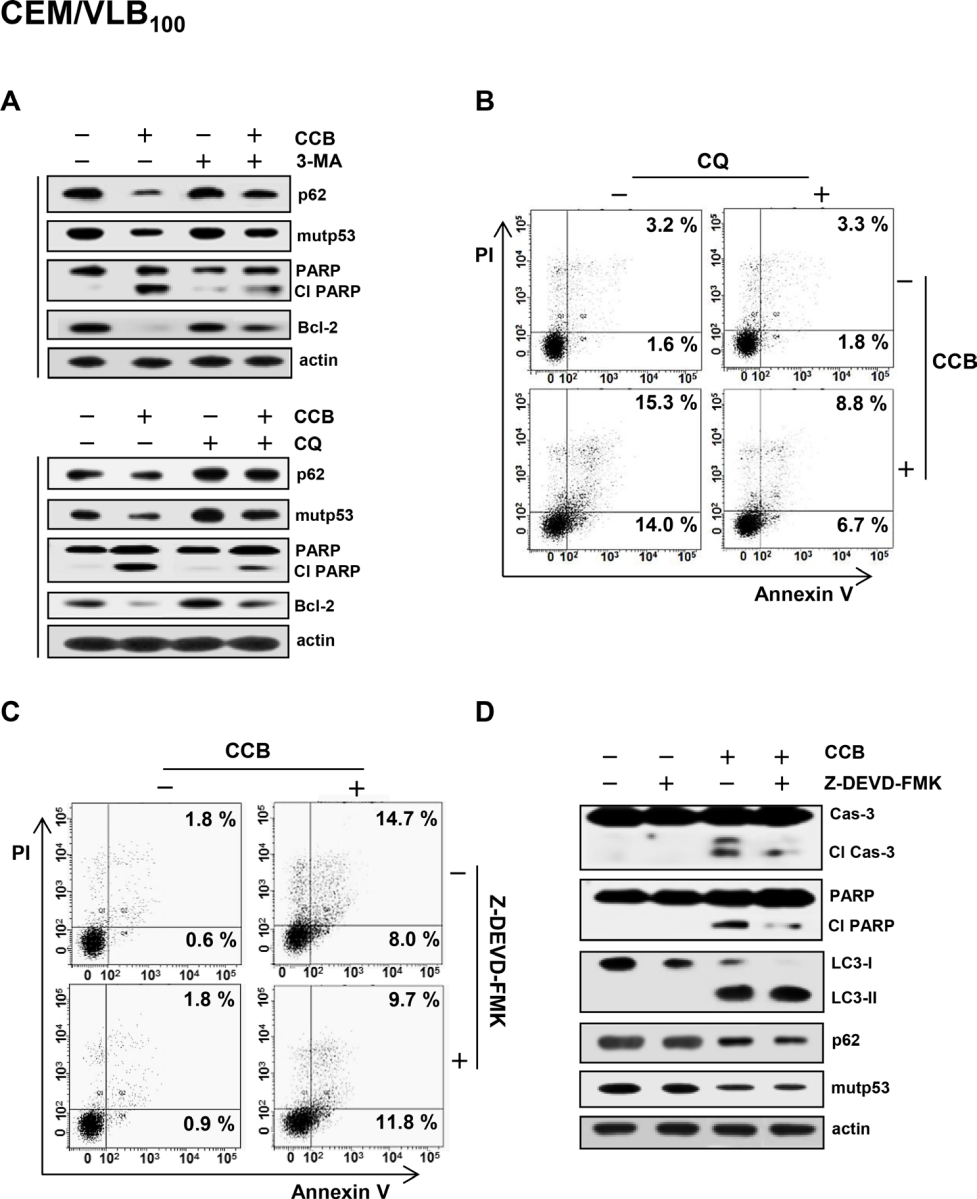
Prevention of CCB-induced autophagic cell death by autophagy inhibitor and crosstalk between CCB-induced apoptotic and autophagic cell death (**A**) CEM/VLB_100_ cells were treated with 25 μM CCB in the absence or presence of 10 mM 3-MA or treated with 20 μM CCB in the absence or presence of 5 μM CQ for 24 h, and levels of p62, mutp53, Bcl-2 and cleaved PARP (Cl PARP) were determined by western blot analysis. CEM/VBL_100_ cells were treated with 20 μM CCB and/or 5 μM CQ (**B**) or 25 μM CCB and/or 25 μM Z-DEVD-FMK (**C**) for 24 h, and the percentages of early and late apoptotic cells were quantified by FACS for Annexin-V and PI staining. Images shown are representative of three independent experiments. (**D**). Levels of cleaved caspase-3 (Cl cas-3) and Cl PARP and changed levels of LC3-I/II, p62 and mutp53 of CEM/VBL_100_ cells treated with 25 μM CCB and/or 25 μM Z-DEVD-FMK for 24 h were determined by western blot analysis.

To further confirm the effect of CCB on autophagic degradation of mutp53, CEM/VLB_100_ cells were treated with CCB in the presence of 3-methyladenine (3-MA), an inhibitor of early stage autophagy, or chloroquine (CQ), an inhibitor of late stage autophagy, and the modulation of CCB-mediated p62 and mutp53 was determined (Figure 5A). Both 3-MA and CQ were found to prevent CCB-induced down-regulation of p62 and mutp53 in MDR cells, suggesting that autophagy is associated with degradation of mutp53. In addition, CCB-induced PARP cleavage and Bcl-2 down-regulation were inhibited by 3-MA or CQ. Based on these results, we hypothesized that CCB-induced apoptosis would be attenuated by autophagy inhibition. After treatment of CEM/VLB_100_ cells with CCB in the absence or presence of CQ, induction of apoptosis was monitored with flow cytometry using propidium iodide and annexin V. CCB-induced apoptosis was significantly reduced by co-treatment with CQ, as determined by summing percentages in the second and fourth quadrants that are considered to be the percentage of apoptotic cells (Figure 5B). These results suggest that autophagy is associated at least in part with CCB-induced cell death. It has also been reported that autophagic cell death is distinct from apoptosis and occurs independently of caspase activity, and thus, may be induced when the apoptosis pathway is blocked [33]. To determine the role of autophagy in CCB-induced cell death, we used caspase-3 inhibitor Z-DEVD-FMK to block apoptosis in CCB-treated CEM/VLB_100_ cells. CEM/VLB_100_ cells were treated with CCB in the presence of Z-DEVD-FMK, and cell death was determined by flow cytometry. Z-DEVD-FMK was not able to prevent CCB-induced apoptotic cell death (Figure 5C), indicating the existence of a caspase-independent type of cell death. Moreover, activation of caspase-3 and PARP cleavage were significantly blocked but LC3-II level was increased and p62 level was reduced in CEM/VLB_100_ cells co-treated with CCB and Z-DEVD-FMK compared with the cells treated with CCB alone (Figure 5D). These results suggest that CCB can induce caspase-3-independent cell death including autophagic cell death.

### Induction of autophagy by CCB via inhibition of the Akt/mTOR and STAT3 signaling pathways

**Figure 6:**
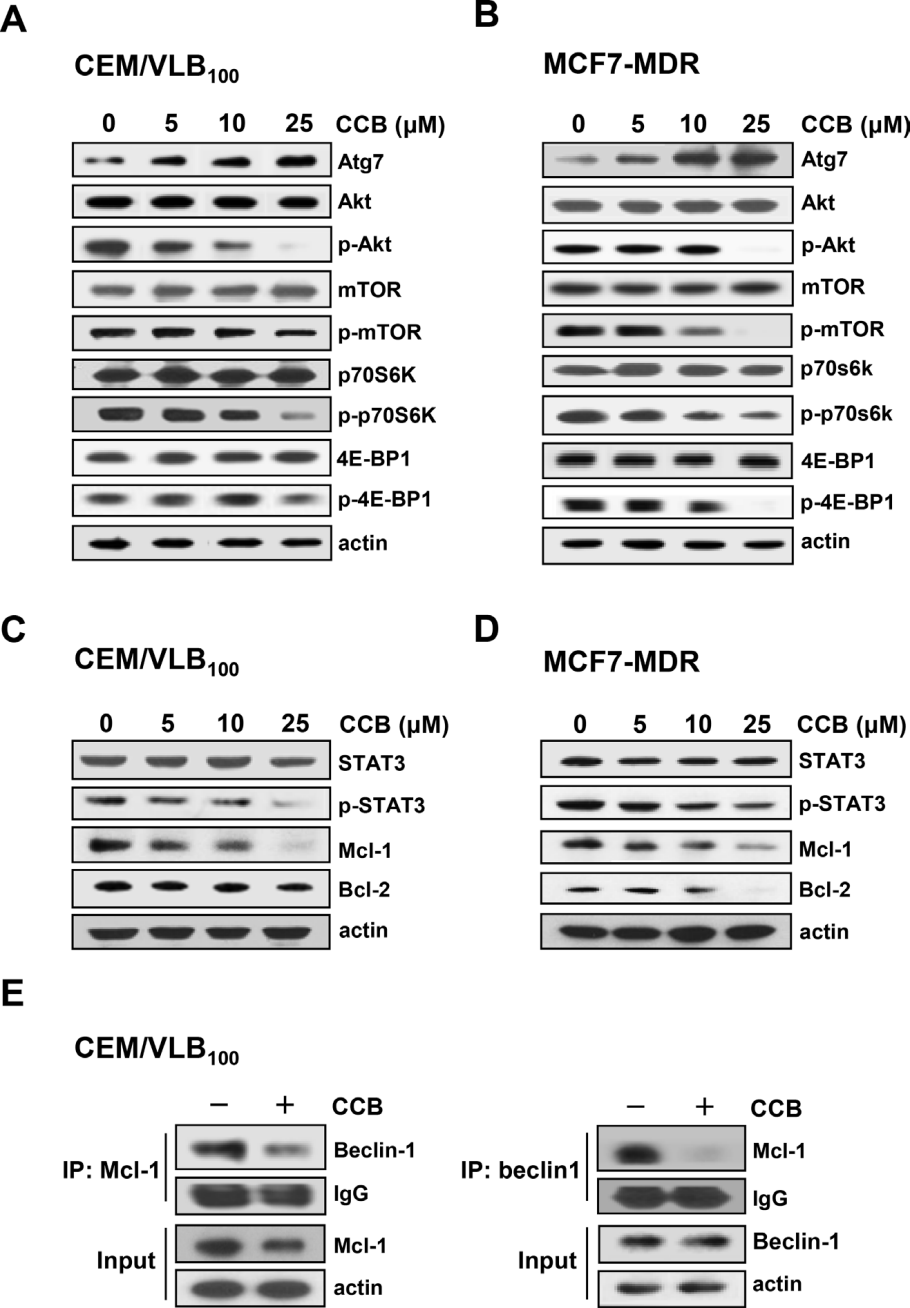
Inhibition of Akt/mTOR signaling pathway and disruption of Beclin-1/Mcl-1 complex via inhibition of STAT3 signaling pathway by CCB in MDR cells CEM/VLB_100_ (**A**) or MCF7-MDR cells (**B**) were treated with increasing doses of CCB for 36 h, and Atg7, Akt/p-Akt, mTOR/p-mTOR, p70S6K/p-p70S6K, and 4E-BP1/p-4E-BP1 levels were determined by western blotting. The expressions of STAT3/p-STAT3 and Mcl-1 in two MDR cells treated with increasing doses of CCB for 36 h (**C** and **D**) were determined by western blot analysis (**E**). Mcl-1 or Beclin-1 were immunoprecipitated from CEM/VLB_100_ cells treated with CCB (25 μM for 36 h) and analyzed for the presence of Mcl-1 or Beclin-1, respectively.

Since Atg7, an essential protein for the induction of autophagy, is required to recruit other proteins to the autophagosomal membrane and to form the autophagic vacuole, and also Akt/mammalian target of rapamycin (mTOR) signaling pathway negatively regulates autophagy [36], we next determined whether CCB could regulate Atg7 expression and phosphorylation of Akt, mTOR, and p70S6K and eukaryotic initiation factor 4E-binding protein (4E-BP1), two downstream effectors of mTOR in CEM/VLB_100_ and MCF7-MDR cells (Figure 6A and 6B) We found that treatment of MDR cells with CCB resulted in a dose-dependent increase in Atg7 expression but decrease in phospho-Akt (p-Akt), phospho-mTOR (p-mTOR), phospho-p70S6K (p-p70S6K) and phospho-4E-BP1(p-4E-BP1) without affecting total levels of them. These results suggest that inhibition of Akt/mTOR/p70S6K/4E-BP1 phosphorylation by CCB participates in the event of CCB-induced autophagy.

Recently, myeloid cell leukemia-1 (Mcl-1), an anti-apoptotic Bcl-2 homolog, has been reported to have a vital role in the regulation of autophagy and also acts as a stress sensor that coordinately controls autophagy and apoptosis [35]. Mcl-1 is known to interact with the autophagy inducing protein Beclin-1, leading to the abrogation of autophagy, and thus signal transducer and activator of transcription 3 (STAT3) mediated-inhibition of autophagy likely occurs through its ability to augment Mcl-1 expression [37]. Since STAT3 regulates the expression of Bcl-2 and Mcl-1, we determined whether CCB could modulate total and phospho-STAT3 (p-STAT3) levels and expression of Bcl-2 and Mcl-1 in MDR cells. In CEM/VLB_100_ and MCF7-MDR cells, CCB significantly reduced p-STAT3 and down-regulated the expression of Mcl-1 and Bcl-2 (Figure 6C and 6D). Furthermore, co-immunoprecipitation studies conducted in CCB-treated CEM/VLB_100_ cells revealed CCB reduced the interaction between Beclin-1 and Mcl-1 without affecting Beclin-1 levels (Figure 6E). These results demonstrated that CCB suppressed p-STAT3 and Mcl-1 and increased Beclin-1 release and thus disrupted Beclin-1/Mcl-1 complex by inhibiting the STAT3 signaling pathway. These observations suggest that CCB induces autophagy by inhibiting the Akt/mTOR signaling pathway and disruption Beclin-1/Mcl-1-complex, and that Akt/mTOR and STAT3 pathways play a role in the regulation of CCB-induced autophagy.

### Inhibition of HSF1 and Hsps and subsequent down-regulation of P-gp expression by NSAIDs

**Figure 7:**
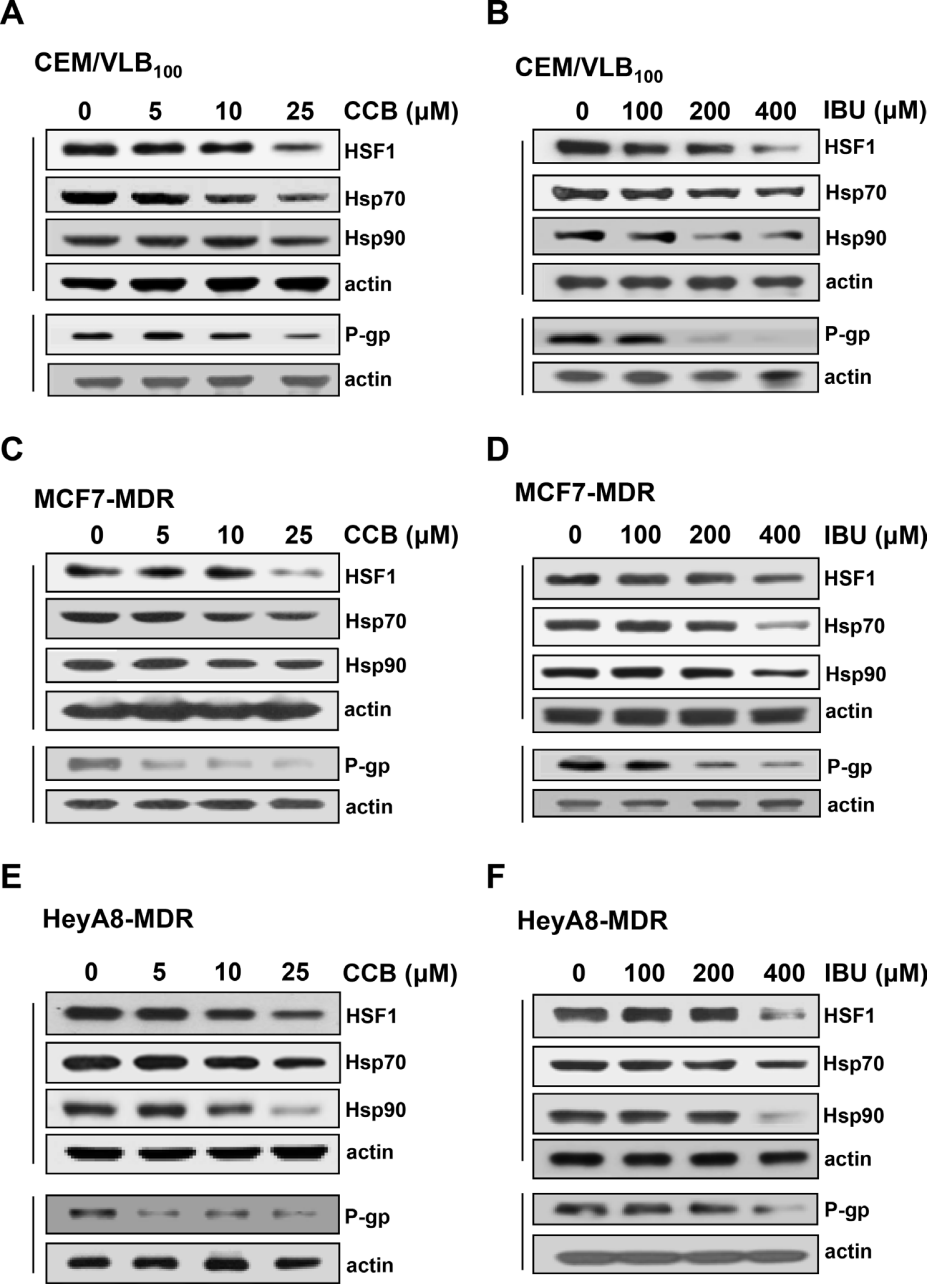
The effects of NSAIDs on HSF1, Hsp70/90 and P-gp levels in MDR cells CEM/VLB_100_ (**A** and **B**), MCF7-MDR (**C** and **D**) or HeyA8-MDR cells (**E** and **F**) were treated with increasing doses of CCB for 36 h (or IBU for 24 h), and changed levels HSF1, Hsp70/90 and P-gp were determined by western blot analysis.

Acquired resistance to Hsp90 inhibitor has been associated with activation of HSF1 and subsequent induction of Hsp70 [12]. Since mutp53 promoted HSF1 activation and up-regulated expression of Hsps [10, 11], and STAT3 inhibition by AG490 lead to HSF1 and Hsp70 reduction in primary effusion lymphoma cells [38]. In addition, both HSF1 and mutp53 selectively up-regulates P-gp through the transcriptional activation of *MDR1* promoter [17, 18]. Therefore, we hypothesized that down-regulation of mutp53 by NASIDs would reduce HSF1/Hsps and subsequent P-gp levels in MDR cells. To define NSAIDs have the ability to modulate the expression of HSF1/Hsps and P-gp, the changes in of HSF1/Hsps and P-gp levels by CCB and IBU were investigated in three MDR cell lines. When CEM/VLB_100_ cells were treated with increasing doses of CCB, the levels of HSF1 and subsequent Hsp70/90 were significantly reduced by treatment of CCB, which caused the down-regulation of P-gp in the MDR cells (Figure 7A). Similarly, the treatment of the MDR cells with IBU resulted in down-regulation of HSF1/Hsps and P-gp in CEM/VLB_100_ cells (Figure 7B). MCF7-MDR and HeyA8-MDR cells also showed down-regulation of HSF1, Hsp70/90 and P-gp by treatment of CCU or IBU (Figure 7C, 7D, 7E and 7F). These results indicate that treatment of MDR cells with NASIDs reduced HSF1/Hsps via inhibition of STAT3 pathway, which caused down-regulation of P-gp and consequently led to sensitization of MDR cells to Hsp90 inhibitors.

### Acceleration of Hsp90 inhibitor-mediated down-regulation of mutp53 and suppression of Hsp90 inhibitor mediated-activation of HSF1, Hsp70 and P-gp by NSAIDs

**Figure 8:**
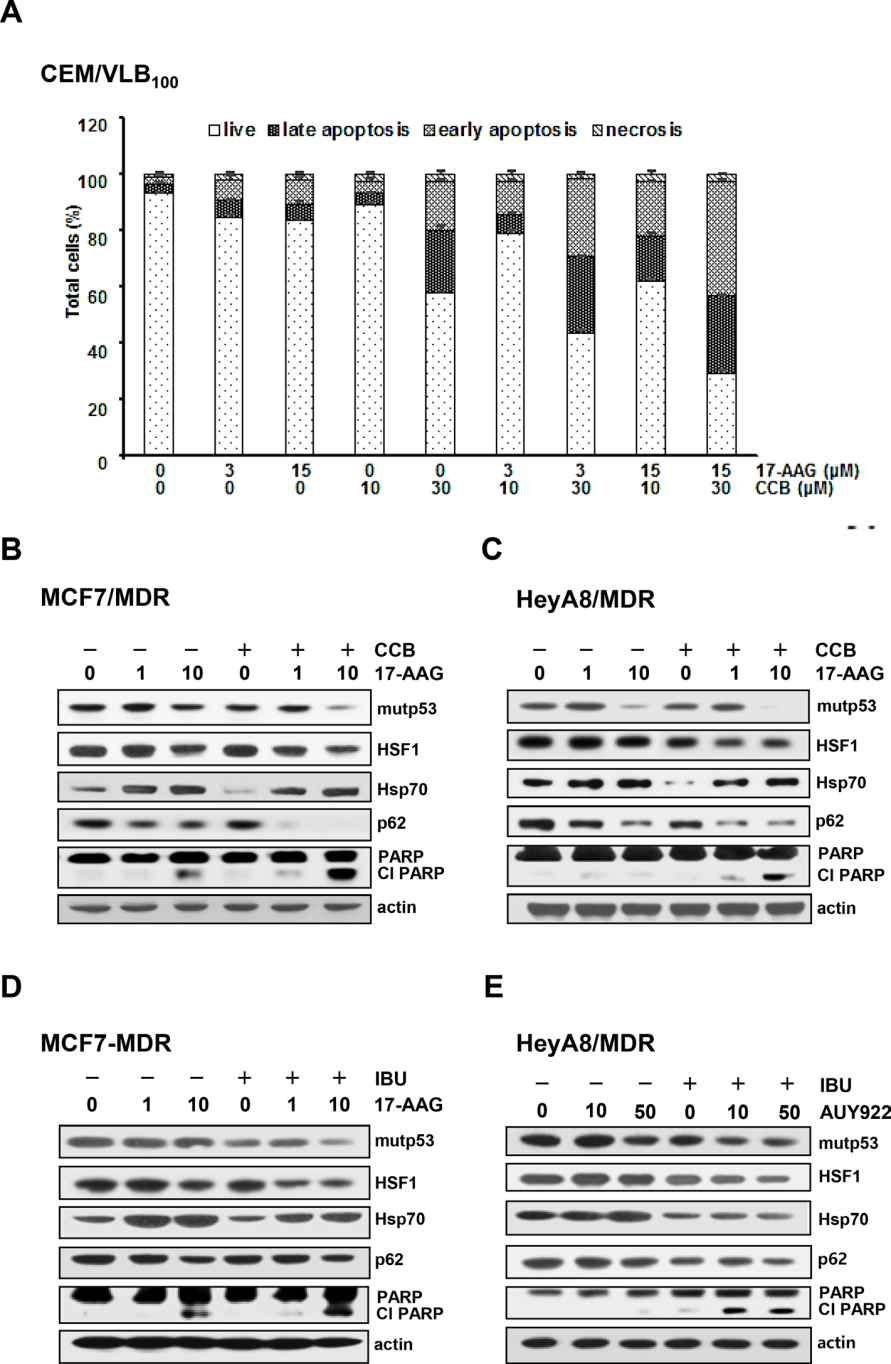
Enhancement of Hsp90 inhibitor-induced apoptosis and acceleration of autophagic mutp53 degradation and suppression of HSF1/Hsp70 activation of MDR cells treated with Hsp90 inhibitor by NSAIDs (**A**) CEM/VBL_100_ cells were treated with 17-AAG (3 or 25 μM) and/or CCB (10 or 30 μM) for 24 h, and the percentages of viable, early apoptosis and late apoptosis/necrosis cells were assessed by flow cytometry with AnV/IP staining. MCF7-MDR (**B** and **D**) and HeyA8-MDR (**C** and **E**) were treated with 17-AAG (1 or 10 μM) or AUY922 (10 or 50 nM) in the presence or absence of 25 μM CCB (or 300 μM IBU) for 24 h. Change levels of mut p53, HSF1/Hsp70, p62 and cleaved PARP were determined by western blot analysis.

**Figure 9:**
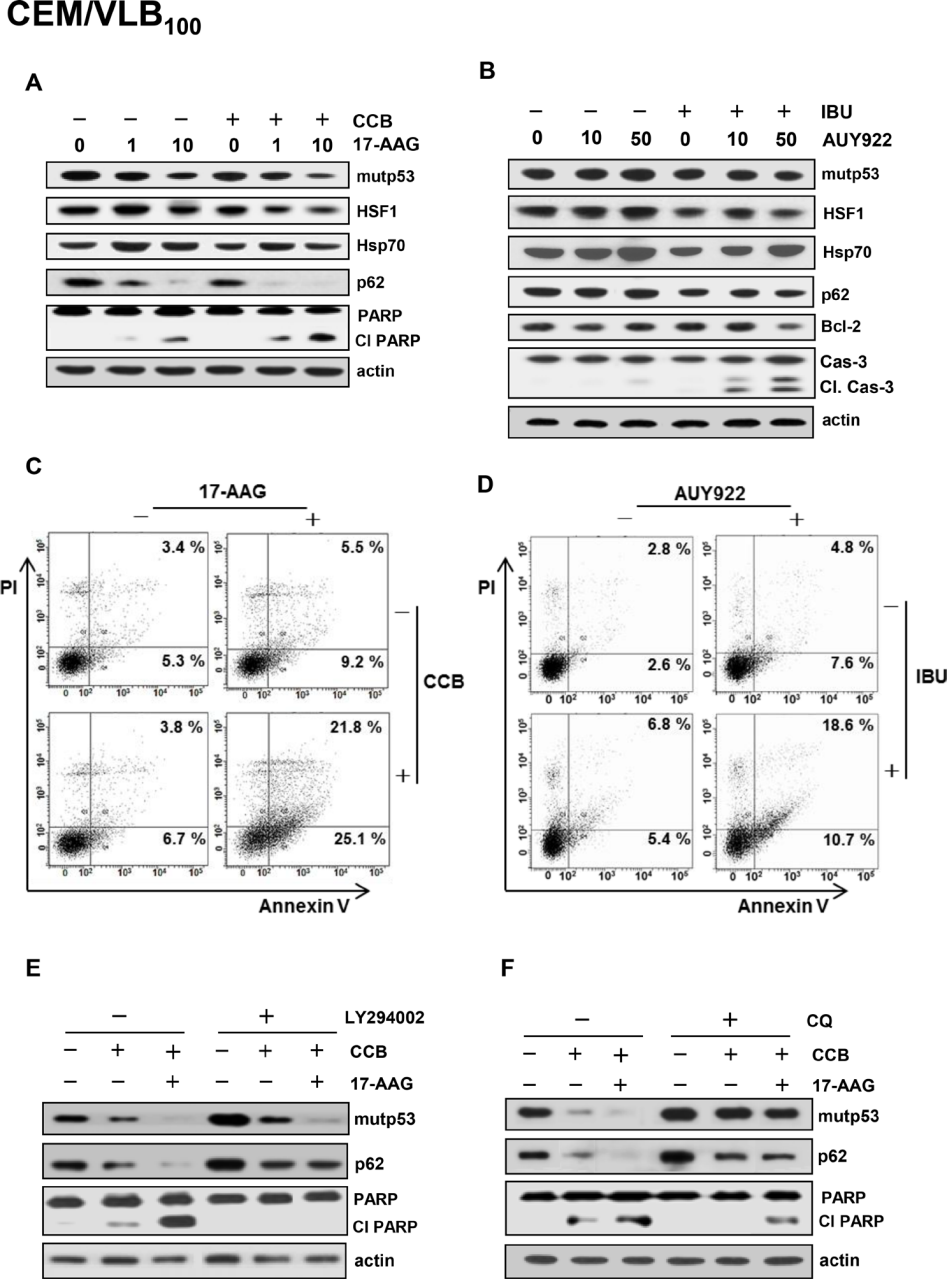
Attenuation of NSAIDs-induced autophagic degradation of mutp53 and apoptosis in MDR cells treated with Hsp90 inhibitor by autophagy inhibition CEM/VBL_100_ cells were co-treated with 17-AAG (1 or 10 μM) 25 μM CCB or AUY922 (10 or 50 nM) in the presence or absence of (or 300 μM IBU) for 24 h, respectively, and changed levels of mut p53, HSF1/Hsp70, p62, Bcl-2, Cl cas-3 and Cl PARP were determined by western blot analysis (**A** and **B**). CEM/VLB_100_ cells were treated with 10 μM 17-AAG and/or 10 μM CCB (**C**) or 50 nM AUY922 and/or 300 μM IBU for 24 h (**D**), and the percentages of early and late apoptotic cells were assessed by flow cytometry with Annexin-V/PI staining. Images shown are representative of three independent experiments. CEM/VLB_100_ cells were co-treated with 25 μM CCB and 10 μM 17-AAG in the presence or absence of 10 μM LY294002 (or 5 μM CQ) for 24 h and changed levels of mutp53, p62 and Cl PARP were determined by western blot analysis (**E** and **F**).

Resistance of MDR cells to Hsp90 inhibitors has been closely linked to up-regulation of P-gp as well as HSF1/Hsps [19]. We therefore hypothesized that down-regulation of mutp53 and HSF1/Hsps and consequent suppression of P-gp function by NASIDs could contribute to sensitize MDR cells to Hsp90 inhibitors and thus reverse Hsp90 inhibitor resistance of the cells. We first determined whether CCB could enhance susceptibility of CEM/VLB_100_ cells to 17-AAG-induced apoptosis using flow cytometry. We observed a dose-dependent sensitization of CEM/VLB_100_ cells to 17-AAG-induced apoptosis by CCB (Figure 8A). Next, we examined whether combination of Hsp90 inhibitor and CCB could modulate Hsp90 inhibitor-mediated changes in mutp53/HSF1/Hsp70 and p62 levels and PARP activity in MCF7-MDR and HeyA8-MDR cells (Figure 8B and 8C). CCB significantly augmented 17-AAG-mediated degradation of mutp53 and suppressed 17-AAG-mediated activation of HSF1 and up-regulation of Hsp70 in these MDR cells. We also determined whether CCB could potentiate Hsp90 inhibitor-mediated reduction of p62 in MDR cells since geldanamycin, one of Hsp90 inhibitors, could induce autophagy by modulating LC3-II and p62 expression [39]. CCB accelerated 17-AAG-mediated reduction of p62 level, indicating that the combined effect of 17-AAG and CCB on the induction of autophagy would accelerate 17-AAG-mediated mutp53 degradation by CCB in these MDR cells. As was expected, PARP cleavage was enhanced by co-treatment with 17-AAG and CCB versus 17-AAG alone. Moreover, IBU also significantly reduced 17-AAG (or AUY922)-mediated mutp53, HSF1/Hsp70, and p62 levels but enhanced PARP cleavage in MCF7-MDR and HeyA8-MDR cells (Figure 8D and 8E). Similarly, CEM/VLB_100_ cells showed that CCB accelerated 17-AAG-mediated down-regulation of p62 and mutp53 degradation and suppressed 17-AAG-mediated activation of HSF1 and up-regulation of Hsp70, resulting in enhanced PARP cleavage (Figure 9A). In addition, the combined effect of IBU and AUY922 on autophagic degradation of mutp53 and suppression of HSF1/Hsp70 was occurred in CEM/VLB_100_ cells, and consequently IBU accelerated AUY922-mediated-caspase 3 activation and Bcl-2 down-regulation (Figure 9B). When the apoptotic effects of 17-AAG, CCB alone, or 17-AAG plus CCB on CEM/VLB_100_ cells were assessed by flow cytometry, it was found that co-treatment of 17-AAG and CCB had a significantly greater effect than treatment with 17-AAG or CCB alone (Figure 9C) Similarly, potentiation of AUY922-induced apoptosis by IBU was observed in CEM/VLB_100_ cells (Figure 9D). These results suggest that down-regulation of Hsp70 and mutp53 by NSAIDs may contribute to sensitize MDR cells to Hsp90 inhibitor-mediated apoptosis. We next determined whether the combined effect of 17-AAG and CCB on autophagy induction and mutp53 degradation could be prevented by inhibiting autophagy. After co-treatment of CEM/VLB_100_ cells with 17-AAG and CCB in the absence or presence of LY294002, an early stage autophagy inhibitor, or CQ, we then examined changes in mutp53 and p62 levels and PARP activity (Figure 9E and 9F). The acceleration of the 17-AAG-induced down-regulation of mutp53 and p62 and PARP activation by CCB were prevented by early- or late- stage autophagy inhibitors, indicating that inhibition of autophagy, either at early or late stage of the process, could block autophagic degradation of mutp53 induced by combined treatment with17-AAG and CCB.

**Figure 10:**
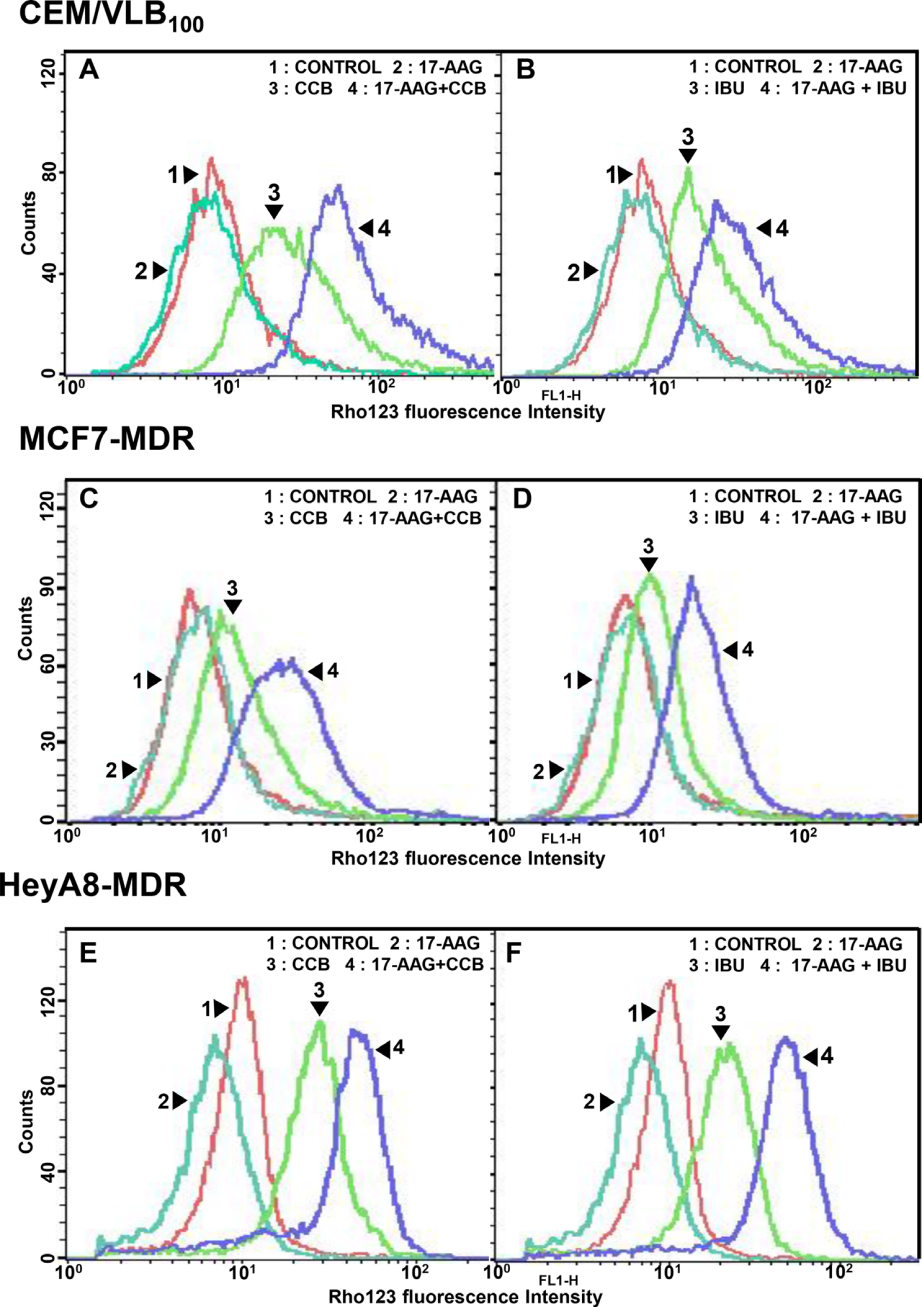
Effect of NSAIDs on P-gp-mediated efflux activity of Hsp90 inhibitors in MDR cells Cell suspensions were isolated from CEM/VBL_100_ (**A** and **B**), MCF7-MDR (**C** and **D**) or HeyA8-MDR cells (**E** and **F**) treated with 5 μM 17-AAG in the presence or absence of CCB (25 μM for 36 h) or IBU (400 μM for 24 h). And then these are incubated with rhodamine 123 (Rho123) and further incubated at 37°C for 4 h to allow P-gp transporter-mediated efflux. Cellular fluorescences were analyzed immediately by flow cytometry.

**Figure 11:**
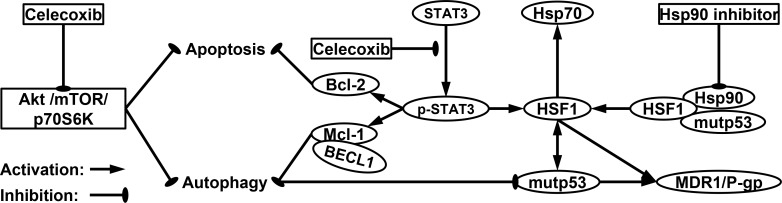
A proposed model of molecular targets of celecoxib in enhancing the cytotoxicity of Hsp90 inhibitors and promoting both apoptotic and autophagic cell death Inhibition of Hsp90 leads to disruption of regulatory complexes of Hsp90 with HSF1 and client protein such as mutp53, thereby causing HSF1-mediated induction of Hsps such as Hsp70 and up-regulation of MDR1/P-gp, which is responsible for the resistance of MDR cells to Hsp90 inhibitor. CCB inhibits phosphorylation of STAT3 that can up-regulate HSF-1, resulting in reduced resistance against Hsp90 inhibitor in MDR cells. Moreover, CCB can promote both apoptotic and autophagic cell death through inhibition of Akt/mTOR/p70S6K pathway and down-regulation of Bcl-2/Mcl-1, which trigger autophagy-mediated degradation of mutp53.

Since P-gp-mediated Hsp90 inhibitor efflux limits the effectiveness of Hsp90 inhibitors [19, 40], we asked whether down-regulation of mutp53 and HSF1/Hsp70 by NSAIDs would effectively block the pump function of Hsp90 inhibitor-mediated P-gp activity. For this purpose, P-gp-mediated transport/efflux was examined using intracellular reduction of fluorescent P-gp substrate Rhodamine 123 (Rho123) determined by flow cytometry in three MDR cell lines treated with 17-AAG or CCB alone versus 17-AAG combined with CCB. In terms of blocking P-gp-mediated Rho123 efflux, co-treatment with CCB and 17-AAG was more effective than each drug alone at blocking P-gp-mediated Rho123 efflux, possibly caused by CCB-induced down-regulation of P-gp in CEM/VLB_100_ cells (Figure 10A). Similarly, IBU and 17-AAG had combination effect in blocking P-gp-mediated Rho123 efflux of CEM/VLB_100_ cells (Figure 10B). We also checked the effect of NSAIDs on Rho123 efflux in MCF7-MDR cells. The efflux of Rho123 in MCF7-MDR cells was prominently blocked by co-treatment of 17-AAG and CCB (or IBU) compared with each drug alone (Figure 10C and 10D). When HeyA8-MDR cells treated with 17-AAG, Rho123 efflux increased, possibly due to the induction of Rho123 efflux by 17-AAG, though this was significantly suppressed by co-treatment of CCB (or IBU) and 17-AAG (Figure 10E and 10F). These results suggest that down-regulation of mutp53/HSF1 and consequent inhibition of P-gp function by NSAIDs may involve sensitization of MDR cells to Hsp90 inhibitors. Hence, we propose that NSAIDs promote both apoptotic and autophagic cell death through inhibition of Akt/mTOR/p70S6K pathway and down-regulation of Bcl-2/Mcl-1, and thus trigger autophagy-mediated degradation of mutp53 and suppress activity of HSF1/Hsp70 and P-gp, eventually leading to sensitization of MDR cells to Hsp90 inhibitors (Figure 11).

## DISCUSSION

Resistance to drug therapy remains a major challenge in cancer chemotherapy. *In vitro* screening of clinical drugs to enhance Hsp90 inhibitor activity in human MDR cancer cells, we found that CCB and IBU, clinically approved NSAIDs, could significantly increase sensitivity of multiple human MDR cancer cell lines toward Hsp90 inhibitors and sensitize the MDR cells to Hsp90 inhibitors, representing NSAIDs as a new class of chemosensitizers for Hsp90 inhibitors. Previously, we reported that amurensin G, a natural SIRT1 inhibitor, enhanced susceptibility of MDR cancer cells to Hsp90 inhibitors, but clinical trial of amurensin G have not been performed yet. NSAIDs, a commonly used class of drugs that act to reduce inflammation, have been tested in clinical trials for its chemopreventive and therapeutic effects against a broad spectrum of human cancers either as a single agent or in combination with other agents [20–25].

We firstly found that NSAIDs-mediated autophagic degradation of mutp53 protein in multiple human MDR cell lines. In MCF7-MDR cells carrying mutp53, CCB consistently reduced mutp53 levels in a dose and time dependent manner but the level of wtp53 protein in CCB-treated parental MCF-7 cells carrying wtp53 was increased under the same conditions, indicating that the regulation of the stability of mutp53 differs from that of wtp53, and mutp53 may be a direct substrate for autophagic degradation. This finding concurs with that of previous study, in which autophagy promoted the destabilization of mutp53 but stabilized wtp53 [13]. Accumulation of mutp53 in MDR cells are known to contribute to resistance to multiple anticancer agents including Hsp90 inhibitors [17, 19]. Thus, prevention of mutp53 accumulation provides an important chemopreventive and chemotherapeutic strategy. Furthermore, Hsp90 is known to be required for the post-translational stability of mutp53, and Hsp90 inhibitors promote the degradation of mutp53 [16]. It is also known that ubiquitin-dependent proteasomal degradation of wtp53 proceeds via the E3-ubiquitin ligase MDM2 [41]. Recently, it has been reported that autophagy is the main route for mutp53 degradation and autophagy controls mutp53 expression levels [42]. Since many p53 mutations have a misfolded configuration and display a high tendency to aggregate, they possess characteristics of typical autophagic substrates. Autophagy protects cells from different types of stress and does not necessarily induce cell death, but on the other side may contribute to cell death (type II programmed cell death, referred to as autophagic cell death). In fact, as autophagy functions to suppress tumors, autophagic activity is often impaired in cancer cells [13]. We present here a novel NSAIDs-induced strategy for killing MDR cells expressing high levels of mutp53 that offers the possibility of reducing elevated mutp53 level in MDR cells. Autophagy may permit cells to survive under unfavorable conditions [31], but paradoxically, stimulation of autophagy in cancer cells could induce cell death, and therefore autophagy, consistent with apoptosis, is also important for the regulation of cancer development and progression and may be regarded as a potential therapeutic approach for anticancer researches [32]. Indeed, rapamycin exhibits an antitumor effect by inducing autophagy in malignant glioma cells and malignant chronic myeloid leukemia, respectively [43]. The functional relationship between autophagic and apoptotic pathways is complex because apoptotic and autophagic response machineries share common inducers, components and this sometimes results in combined autophagy and apoptosis [44], which raises the possibility of developing anti-cancer strategies based on the synergistic modulations of autophagy and apoptosis. Autophagic cell death occurs independently of caspase activity and may be induced when the apoptosis pathway is inhibited. In the present study, treatment of MDR cells with Z-DEVD-FMK (a caspase-3 inhibitor) did not restore cell viability and failed to suppress CCB-induced cell death completely, indicating that apoptosis induced by CCB is partially regulated by a caspase-3 independent, autophagy-based cell death mechanism in MDR cells. In addition, CCB-induced autophagic cell death accelerated 17-AAG-induced autophagy, and subsequent mutp53 down-regulation by CCB were blocked by pretreating MDR cells with autophagy inhibitor, which indicates autophagy inhibition prevents the autophagic degradation of mutp53 by treatment of CCB alone or combined treatment of CCB with Hsp90 inhibitor. As a response to anticancer treatments, whether autophagy activation leads to cell survival or cell death remains controversial, and also autophagy has a dual role in MDR tumors, having tumor-promoting and tumor-suppressing properties [41]. In the present study, NSAIDs induced autophagy in multiple MDR cells since treatment of MDR cells with NSAIDs such as CCB and IBU led to a dose-dependent increase in LC3-II level and decrease in p62 level that plays a central role in autophagy-associated cell death, which also contributed to potentiate Hsp90 inhibitor-induced cell death of MDR cells by NSAIDs. NASIDs induced mutp53 degradation of MDR cells through autophagy in COX-2 inhibition and COX-2-unrelated pathways since COX-2 inactive analogue DMC also induced autophagic degradation of mutp53. Indeed, a number of selective COX-2 inhibitors involving CCB possessed many additional targets besides COX-2 through which they exert their antitumor activities [33]. Therefore, we propose a molecular mechanism whereby COX-2 inhibition and COX-2-unrelated pathways cause the degradation/down-regulation of mutp53 in MDR cells, which rendered MDR cells sensitive to Hsp90 inhibitor-mediated cell death.

We present that inhibition of Akt/mTOR/p70S6K/4E-BP1 phosphorylation participates in the molecular events of CCB-induced autophagy. Moreover, CCB induced down-regulation of p-STAT3 and Mcl-1, a downstream target of STAT3, and caused to release of Beclin-1 and disruption of the Beclin-1/Mcl-1 complex, and it could contribute to induce autophagy in MDR cells because Mcl-1-dependent activation of Beclin-1 through disruption of the Beclin-1/Mcl-1 complex mediates autophagic cell death [37]. Mcl-1 plays a key role in determining cell fate by coordinately regulating apoptosis and autophagy. Indeed, in a previous study, CCB induced apoptosis by inhibiting the expressions of p-STAT3 and Mcl-1 in nasopharyngeal carcinoma cells [45]. Therefore, inactivation of STAT3 and subsequent down-regulation of Mcl-1 by NSAIDs might be a key event in the activation of apoptosis and autophagy in MDR cells. The Hsp90 inhibitor-induced activation of HSF1 and up-regulation Hsp70, major determinants of Hsp90 inhibitor resistance, might be modulated by the expression of Mcl-1 through STAT3 signaling [37]. Indeed, down-regulation of the expression of HSF1/Hsp70 by STAT3 inhibitor AG490 suppressed Mcl-1 and led to the induction of apoptosis and autophagy in primary effusion lymphoma cells [38]. The present study shows that CCB-mediated down-regulation of STAT3/Mcl-1 was associated with down-regulation of HSF1 and Hsp70/90 in MDR cells, suggesting down-regulation of Hsps by NSAIDs leads to induction of autophagy as a compensatory mechanism to maintain cell integrity. Therefore, our results suggest that regulation of Akt/mTOR and STAT3/Mcl-1 pathways plays a major role in activation of autophagy and apoptosis in MDR cells. As mutp53 is an important determinant of HSF1 activity, HSF1 is specifically up-regulated by mutp53 but not wtp53 [11] and also controls the stability of mutp53 protein in human cancer cells via activation of Hsp90, which strongly stabilizes mutp53 [41], indicating cross-talk between HSF1 and mutp53. Moreover, up-regulation of HSF1 and mutp53 could increase *MDR1*/P-gp expression in MDR cells [17, 18], and thus, inhibition of HSF1/Hsps and mutp53 by NSAIDs might contribute to the overcoming of Hsp90 inhibitor resistance in MDR cells [19]. It has been reported that IBU inhibits the expression of Hsp70 by down-regulating HSF1 in human lung cancer A549 cells [26]. Our data show that NSAIDs including CCB and IBU have the ability to down-regulate HSF1/Hsps, and thus, to reverse Hsp90 inhibitor resistance in MDR cells. Knockdown of HSF1 significantly promoted starvation-induced autophagy and conversely overexpression of Hsp70 significantly blocked this autophagy induction in cell culture model [46], indicating involvement of HSF1/Hsp70 expression in autophagy regulation. Our results indicated that down-regulation of HSF1/Hsps by NSAIDs was involved in autophagy induction in MDR cells, which is possibly aimed at the elimination of the accumulating unfolded proteins such as mutp53. To the best of our knowledge, this is the first study to demonstrate that NSAIDs induce autophagy and promote the apoptotic and autophagic cell deaths of MDR cells treated with Hsp90 inhibitor, and that NSAIDs significantly attenuated Hsp90 inhibitor-mediated upregulation of HSF1/Hsp70 levels and accelerated Hsp90 inhibitor-mediated of p62 reduction, mutp53 degradation and PARP cleavage, suggesting that NSAIDs can increase the cytotoxicity of Hsp90 inhibitors via augmentation of autophagic and apoptotic pathways. It has been reported that over-expression of P-gp is a possible mechanism of acquired resistance to Hsp90 inhibitors [12, 19, 40], and thus P-gp inhibitors might offer a means of overcoming Hsp90 inhibitor resistance caused by P-gp over-expression. Our data showed that co-treatment of CCB (or IBU) and 17-AAG resulted in suppression of P-gp pump function through blocking P-gp-mediated efflux of Rho123 more effectively than CCB (or IBU) alone in multiple MDR cell lines, indicating that NSAIDs-induced potentiation of Hsp90 inhibitor cytotoxicity in MDR cells depends on down-regulation of mutp53/HSF1/P-gp signaling cascade.

Taken together, our results have demonstrated that NSAIDs promote both apoptotic and autophagic cell death through inhibition of Akt/mTOR/p70S6K pathway and down-regulation of Bcl-2/Mcl-1, and consequently trigger autophagy-mediated degradation of mutp53 and suppress Hsp90 inhibitor-induced activation of HSF1/Hsp70 and P-gp, causing sensitization of MDR cells to Hsp90 inhibitors. Therefore, CCB and IBU can be considered as chemosensitizers of Hsp90 inhibitor for effective treatment of MDR cancers.

## MATERIALS AND METHODS

### Cell culture and reagents

Three human MDR cell-line variants, MCF7-MDR cells isolated from human breast cancer MCF-7 cells, CEM/VLB_100_ cells isolated from CEM human lymphoblastic leukemia cells and HeyA8-MDR cells isolated from HeyA8 human ovarian cancer cells were used in this study. These cells were maintained in DMEM (MCF7-MDR and HeyA8-MDR cells) or RPMI1640 (CEM/VLB_100_ cells) supplemented with 10% fetal bovine serum and were incubated at 37°C, 5% CO_2_ and 95% humidity. 17-allylamino-17-demethoxy-geldanamycin (17-AAG) and NVP-AUY922 were purchased from Enzo Life Sciences Inc. (Farmingdale, NY, USA) and Selleck Chemicals (Houston, TX, USA), respectively. Celecoxib (CCB), (S)-(+)-ibuprofen (ibuprofen, IBU), cycloheximide (CHX), chloroquine (CQ), 3-methyladenine (3MA), 2, 5-dimethyl-celecoxib (DMC) and LY294002 were purchased from Sigma-Aldrich (St. Louis, MO, USA). Z-DEVD-FMK was purchased from R&D Systems (Minneapolis, MN, USA).

### Cell proliferation assay

The effects of Hsp90 inhibitor and/or NSAIDs on cell proliferation were determined using a 3-(4,5-dimethylthiazol-2-yl)-2,5-diphenyltetrazolium bromide (MTT) assay. Exponentially growing MDR cells were plated in a 96-well plate and treated with 17-AAG or AUY922 in the presence or absence of CCB or IBU, at 37°C for 96 h. Growth inhibitions of drug-treated MDR cells were expressed as percentages of untreated controls. At least two separate experiments were performed in triplicate. Interactions between 17-AAG (or AUY922) and CCB (or IBU) were assessed using the Compu-Syn Software program (ComboSyn, Paramus, NJ, USA), which utilizes the Chou-Talalay equation. A combination index (CI) of < 0.9 represents drug synergism, of 0.9 to < 1.1 near additive effects, and of ≥ 1.1 antagonism. All experiments were carried out in triplicate.

### Western blot and co-immunoprecipitation analysis

Cell lysates from control and drug-treated cells were prepared using M-PER Reagent (Thermo Scientific Inc., USA). Lysates were clarified by centrifugation, and equal amounts of proteins were resolved by SDS-PAGE and immunoblotted with indicated antibodies. Western blot analysis was performed using specific primary antibodies against LC3 and p62 (Novus Biologicals, Littleton, CO, USA), Atg7, Beclin-1, Mcl-1, STAT3, phospho-STAT3 (Tyr705) Akt, phospho-Akt (Thr308), mTOR, phospho-mTOR (Ser2448), 4E-BP1, phospho-4E-BP1 (Thr37/46), p70S6K, phospho-p70S6K (Thr389) and caspase-3 (Cell Signaling, Danvers, MA, USA), p53, PARP, Bcl-2, Bax, HSF1, P-gp (Santa Cruz Biotechnology, CA, USA), Hsp70, Hsp90 (Enzo Life Sciences, NY, USA) and b-actin (Sigma-Aldrich, St. Louis, MO, USA). The p53 antibody (DO-1) used was a mouse monoclonal antibody raised against amino acids 11-25 of p53 of human origin, which was recommended for detection of wild and mutant human p53. For co-immunoprecipitation, whole cell extracts from CEM/VLB_100_ cells treated with or without CCB were incubated with indicated antibodiesovernight at 4°C. Protein G-Sepharose beads containing immunocomplexes were boiled, electrophoresed on 8% SDS-polyacrylamide gels, and analyzed by western blotting using indicated antibodies.

### Apoptosis assessment by Annexin V staining

Cells were treated with Hsp90 inhibitor in the presence or absence of NSAIDs or autophagy/apoptosis inhibitor under indicated conditions, centrifuged and resuspended in 100 μl of the staining solution containing Annexin V-fluorescein (FITC Apoptosis detection kit; BD ParMingen San Diego, CA, USA) and propidium iodide (Sigma-Aldrich, St. Louis, MO, USA) in a Hepes buffer. After incubation at room temperature for 20 min, the percentage of early (annexin V positive/PI negative) and late apoptotic cells (annexin V positive/PI positive) was quantified by FACS for Annexin-V and PI staining.

### Flow-cytometric dye-efflux assay

Cell suspension (500 µl) of MDR cells treated with 17-AAG (or AUY922) and/or CCB (or IBU) were incubated with 0.5 µg/ml rhodamine 123 (Rho123; a fluorescent substrate of P-gp) at 37°C for 30 min, washed with ice-cold PBS, and further incubated at 37°C for 3 h to allow P-gp-mediated drug efflux. Cells were pelleted by centrifugation at 500×g and resuspended in PBS. Cellular fluorescence was analyzed immediately using a FACS flow cytometer (FACScalibur, BD Biosciences, San Jose, CA, USA).

### Statistical analysis

The results obtained were expressed as the mean ± S.E. of at least three independent experiments. A Student’s *t*-test was used to calculate the statistical significance of the experimental data and the level of significance was set as ^*^p < 0.05, ^**^p < 0.01 and ^***^p < 0.001.

## References

[1] Wang Y, Koay YC, McAlpine SR (2017). How selective are Hsp90 inhibitors for cancer cells over normal cells?. ChemMedChem.

[2] Haque A, Alam Q, Alam MZ, Azhar EI, Sait KHW, Anfinan N, Mushtaq G, Kamal MA, Rasool M (2016). Current understanding of Hsp90 as a novel therapeutic target : An emerging approach for the treatment of cancer. Curr Pharm Des.

[3] Hendriks LEL, Dingemans AMC (2017). Heat shock protein antagonists in early stage clinical trials for NSCLC. Expert Opin Investig Drugs.

[4] Sidera K, Patsavoudi E (2014). Hsp90 inhibitors : Current development and potential in cancer therapy. Recent Patents Anticancer Drug Discov.

[5] Tatokoro M, Koga F, Yoshida S, Kihara K (2015). Heat shock protein 90 targeting therapy : State of the art and future perspective. Excli J.

[6] Bendell JC, Bauer TM, Lamar R, Joseph M, Penley W, Thompson DS, Spigel DR, Owera R, Lane CM, Earwood C, Burris HA (2016). A phase 2 study of the Hsp90 inhibitor AUY922 as treatment for patients with refractory gastrointestinal stromal tumors. Cancer Invest.

[7] Lian JP, Lin DQ, Xie X, Xu YZ, Xu LY, Meng L, Zhu Y (2017). NVP-AUY922, a novel Hsp90 inhibitor, inhibits the progression of malignant pheochromocytoma *in vitro* and *in vivo*. Onco Targets Ther.

[8] Pedersen KS, Kim GP, Foster NR, Wang-Gillam A, Erlichman C, McWilliams RR (2015). Phase II trial of gemcitabine and tanespimycin (17AAG) in metastatic pancreatic cancer : a Mayo Clinic Phase II Consortium study. Invest New Drugs.

[9] Weber H, Valbuena JR, Barbhuiya MA, Stein S, Kunkel H, Garcia P, Bizama C, Riquelme I, Espinoza JA, Kurtz SE, Tyner JW, Calderon JF, Corvalan AH (2017). Small molecule inhibitor screening identifified Hsp90 inhibitor 17-AAG as potential therapeutic agent for gallbladder cancer. Oncotarget.

[10] Alexandrova EM, Marchenko ND (2015). Mutant p53 - heat shock response oncogenic cooperation : A new mechanism of cancer cell survival. Front Endocrinol (Lausanne).

[11] Li D, Yallowitz A, Ozog L, Marchenko N (2014). A gain-of-function mutant p53-HSF1 feed forward circuit governs adaptation of cancer cells to proteotoxic stress. Cell Death Dis.

[12] Piper PW, Millson SH (2011). Mechanisms of resistance to Hsp90 inhibitor drugs: A complex mosaic emerges. Pharmaceuticals (Basel).

[13] Choudhury S, Kolukula VK, Preet A, Albanese C, Avantaggiati ML (2013). Dissecting the pathways that destabilize mutant p53 the proteasome or autophagy?. Cell Cycle.

[14] Morton JP, Timpson P, Karim SA, Ridgway RA, Athineos D, Doyle B, Jamieson NB, Oien KA, Lowy AM, Brunton VG, Frame MC, Evans TRJ, Sansom OJ (2010). Mutant p53 drives metastasis and overcomes growth arrest/senescence in pancreatic cancer. Proc Natl Acad Sci USA.

[15] Petitjean A, Achatz MIW, Borresen-Dale AL, Hainaut P, Olivier M (2007). TP53 mutations in human cancers : functional selection and impact on cancer prognosis and outcomes. Oncogene.

[16] Muller P, Hrstka R, Coomber D, Lane DP, Vojtesek B (2008). Chaperone-dependent stabilization and degradation of p53 mutants. Oncogene.

[17] Sampath J, Sun DX, Kidd VJ, Grenet J, Gandhi A, Shapiro LH, Wang QJ, Zambetti GP, Schuetz JD (2001). Mutant p53 cooperates with ETS and selectively up-regulates human MDR1 not MRP1. J Biol Chem.

[18] Vilaboa NE, Galan A, Troyano A, de Blas E, Aller P (2000). Regulation of multidrug resistance 1 (MDR1)/P-glycoprotein gene expression and activity by heat-shock transcription factor 1 (HSF1). J Biol Chem.

[19] Kim HB, Lee SH, Um JH, Oh WK, Kim DW, Kang CD, Kim SH (2015). Sensitization of multidrug-resistant human cancer cells to Hsp90 inhibitors by down-regulation of SIRT1. Oncotarget.

[20] Diaz-Gonzalez F, Sanchez-Madrid F (2015). NSAIDs: Learning new tricks from old drugs. Eur J Immunol.

[21] Harris RE, Beebe J, Alshafie GA (2012). Reduction in cancer risk by selective and nonselective cyclooxygenase-2 (COX-2) inhibitors. J Exp Pharmacol.

[22] Hilovska L, Jendzelovsky R, Fedorocko P (2015). Potency of non-steroidal anti-inflammatory drugs in chemotherapy. Mol Clin Oncol.

[23] Umar A, Steele VE, Menter DG, Hawk ET (2016). Mechanisms of nonsteroidal anti-inflammatory drugs in cancer prevention. Semin Oncol.

[24] Jendrossek V (2013). Targeting apoptosis pathways by Celecoxib in cancer. Cancer Lett.

[25] Liu R, Xu KP, Tan GS (2015). Cyclooxygenase-2 inhibitors in lung cancer treatment: Bench to bed. Eur J Pharmacol.

[26] Endo H, Yano M, Okumura Y, Kido H (2014). Ibuprofen enhances the anticancer activity of cisplatin in lung cancer cells by inhibiting the heat shock protein 70. Cell Death Dis.

[27] Dharmapuri G, Doneti R, Philip GH, Kalle AM (2015). Celecoxib sensitizes imatinib-resistant K562 cells to imatinib by inhibiting MRP1–5, ABCA2 and ABCG2 transporters via Wnt and Ras signaling pathways. Leuk Res.

[28] Takara K, Hayashi R, Kokufu M, Yamamoto K, Kitada N, Ohnishi N, Yokoyama T (2009). Effects of nonsteroidal anti-inflammatory drugs on the expression and function of P-glycoprotein/MDR1 in Caco-2 cells. Drug Chem Toxicol.

[29] Xu HB, Shen FM, Lv QZ (2015). Celecoxib enhanced the cytotoxic effect of cisplatin in drug-resistant human gastric cancer cells by inhibition of cyclooxygenase-2. Eur J Pharmacol.

[30] Zrieki A, Farinotti R, Buyse M (2010). Cyclooxygenase-2 inhibitors prevent trinitrobenzene sulfonic acid-induced P-glycoprotein up-regulation *in vitro* and *in vivo*. Eur J Pharmacol.

[31] Barth S, Glick D, Macleod KF (2010). Autophagy : assays and artifacts. J Pathol.

[32] Sui X, Chen R, Wang Z, Huang Z, Kong N, Zhang M, Han W, Lou F, Yang J, Zhang Q, Wang X, He C, Pan H (2013). Autophagy and chemotherapy resistance: a promising therapeutic target for cancer treatment. Cell Death Dis.

[33] Cervello M, Bachvarov D, Cusimano A, Sardina F, Azzolina A, Lampiasi N, Giannitrapani L, McCubrey JA, Montalto G (2011). COX-2-dependent and COX-2-independent mode of action of celecoxib in human liver cancer cells. OMICS.

[34] Schonthal AH (2010). Exploiting cyclooxygenase-(in)dependent properties of COX-2 inhibitors for malignant glioma therapy. Anticancer Agents Med Chem.

[35] Bushra R, Aslam N (2010). An overview of clinical pharmacology of Ibuprofen. Oman Med J.

[36] Viola G, Bortolozzi R, Hamel E, Moro S, Brun P, Castagliuolo I, Ferlin MG, Basso G (2012). MG-2477, a new tubulin inhibitor, induces autophagy through inhibition of the Akt/mTOR pathway and delayed apoptosis in A549 cells. Biochem Pharmacol.

[37] Tai WT, Shiau CW, Chen HL, Liu CY, Lin CS, Cheng AL, Chen PJ, Chen KF (2013). Mcl-1-dependent activation of Beclin 1 mediates autophagic cell death induced by sorafenib and SC-59 in hepatocellular carcinoma cells. Cell Death Dis.

[38] Granato M, Chiozzi B, Filardi MR, Lotti LV, Di Renzo L, Faggioni A, Cirone M (2015). Tyrosine kinase inhibitor tyrphostin AG490 triggers both apoptosis and autophagy by reducing HSF1 and Mcl-1 in PEL cells. Cancer Lett.

[39] Mori M, Hitora T, Nakamura O, Yamagami Y, Horie R, Nishimura H, Yamamoto T (2015). Hsp90 inhibitor induces autophagy and apoptosis in osteosarcoma cells. Int J Oncol.

[40] Zhang H, Neely L, Lundgren K, Yang YC, Lough R, Timple N, Burrows F (2010). BIIB021, a synthetic Hsp90 inhibitor, has broad application against tumors with acquired multidrug resistance. Int J Cancer.

[41] Li D, Marchenko ND, Schulz R, Fischer V, Velasco-Hernandez T, Talos F, Moll UM (2011). Functional inactivation of endogenous MDM2 and CHIP by HSP90 causes aberrant stabilization of mutant p53 in human cancer cells. Mol Cancer Res.

[42] Garufi A, Pucci D, D’Orazi V, Cirone M, Bossi G, Avantaggiati ML, D’Orazi G (2014). Degradation of mutant p53H175 protein by Zn(II) through autophagy. Cell Death Dis.

[43] Takeuchi H, Kondo Y, Fujiwara K, Kanzawa T, Aoki H, Mills GB, Kondo S (2005). Synergistic augmentation of rapamycin-induced autophagy in malignant glioma cells by phosphatidylinositol 3-kinase/protein kinase B inhibitors. Cancer Res.

[44] Maiuri MC, Zalckvar E, Kimchi A, Kroemer G (2007). Self-eating and self-killing : crosstalk between autophagy and apoptosis. Nat Rev Mol Cell Biol.

[45] Liu DB, Hu GY, Long GX, Qiu H, Mei Q, Hu GQ (2012). Celecoxib induces apoptosis and cell-cycle arrest in nasopharyngeal carcinoma cell lines via inhibition of STAT3 phosphorylation. Acta Pharmacol Sin.

[46] Dokladny K, Zuhl MN, Mandell M, Bhattacharya D, Schneider S, Deretic V, Moseley PL (2013). Regulatory coordination between two major intracellular homeostatic systems heat shock response and autophagy. J Biol Chem.

